# How to analyse and manipulate nonlinear phenomena in voice recordings

**DOI:** 10.1098/rstb.2024.0003

**Published:** 2025-04-03

**Authors:** Andrey Anikin, Christian T. Herbst

**Affiliations:** ^1^ Division of Cognitive Science, Lund University, Lund, Sweden; ^2^ ENES Bioacoustics Research Laboratory, Université Jean Monnet Saint-Étienne, Saint-Étienne, France; ^3^ University of Vienna, Vienna, Austria; ^4^ Department of Communication Sciences and Disorders, College of Liberal Arts and Sciences, University of Iowa, Iowa City, Iowa, USA

**Keywords:** voice, vocal communication, nonlinear phenomena, acoustic analysis, voice synthesis

## Abstract

We address two research applications in this methodological review: starting from an audio recording, the goal may be to characterize nonlinear phenomena (NLP) at the level of voice production or to test their perceptual effects on listeners. A crucial prerequisite for this work is the ability to detect NLP in acoustic signals, which can then be correlated with biologically relevant information about the caller and with listeners’ reaction. NLP are often annotated manually, but this is labour-intensive and not very reliable, although we describe potentially helpful advanced visualization aids such as reassigned spectrograms and phasegrams. Objective acoustic features can also be useful, including general descriptives (harmonics-to-noise ratio, cepstral peak prominence, vocal roughness), statistics derived from nonlinear dynamics (correlation dimension) and NLP-specific measures (depth of modulation and subharmonics). On the perception side, playback studies can greatly benefit from tools for directly manipulating NLP in recordings. Adding frequency jumps, amplitude modulation and subharmonics is relatively straightforward. Creating biphonation, imitating chaos or removing NLP from a recording are more challenging, but feasible with parametric voice synthesis. We describe the most promising algorithms for analysing and manipulating NLP and provide detailed examples with audio files and R code in supplementary material.

This article is part of the theme issue ‘Nonlinear phenomena in vertebrate vocalizations: mechanisms and communicative functions’.

## 1. Introduction

**Figure 1.**
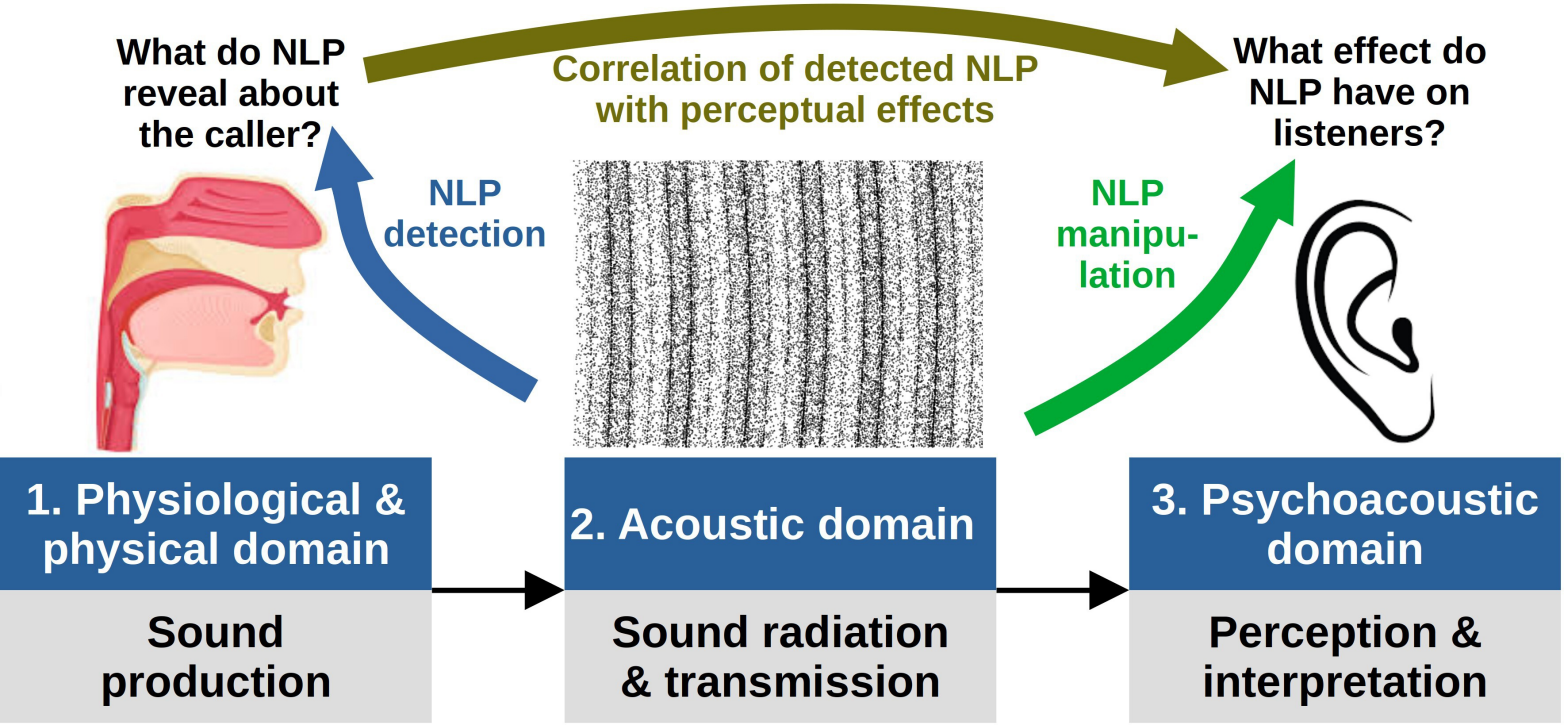
Brunswik’s lens model of communication [5] applied to NLP research. The middle panel represents acoustic waves propagating through the air, with the density of dots corresponding to local pressure fluctuations. The sound is a 100 Hz synthetic steady tone, amplitude modulated at 30 Hz with a modulation depth of 40% (both frequencies are visible as dark bands). See text for further explanation.

Nonlinear phenomena (NLP) is an umbrella term for certain oscillatory states of the vertebrate sound generation system, or for transitions (bifurcations) between these states. They encompass phenomena such as frequency jumps, subharmonics, deterministic chaos and biphonation (see [1,2] in this volume for an overview of NLP classifications). The various NLP can be considered on three levels: (i) as oscillatory features of the voice production apparatus; (ii) as features of the radiated acoustic voice signal; and (iii) as phenomena that evoke certain sensory impressions and behavioural effects on the perceiving end of vocal communication. The study of NLP is a vast, technically demanding research domain. To limit the scope of this review and to keep it relevant for applied research in bioacoustics and psychology, we focus on the acoustic domain and provide methodological guidelines and tools for answering two main research questions:

*Question 1: what do NLP reveal about the caller?* Here, the ultimate objective is to learn what biologically relevant information is available in the signal due to the presence of NLP. For example, we may be interested in whether NLP encode information about the age, health, mate quality or motivational and affective state of the caller (e.g. [3,4]). Answering this question requires knowledge of the physiological reality of NLP production. Because this information is not directly observable, a variety of techniques are employed to infer it. Specifically, the task we focus on here is detecting NLP from a recorded acoustic signal (the blue path in figure 1). We also list the main methods for investigating voice production more directly, such as with high-speed imaging *in vivo* or via excised larynx experimentation (see electronic supplementary material, table S1, for a list of key resources, including particularly useful algorithms and measures, and [6,7] for recent reviews), but do not cover them in detail because these methods are not widely available outside clinical voice science. Direct *in vivo* observation of voice production is particularly challenging in non-human animals, and empirical evidence is very sparse. Instead, we assume that researchers will normally only have access to audio recordings, although many of the discussed analytical techniques are also applicable to physiological signals such as electroglottographic (EGG) recordings [8]. Likewise, computational simulation methods—while very useful for creating biophysical models of phonation and understanding NLP at a fundamental level—are less relevant to NLP detection in audio recordings, and they are reviewed elsewhere [9,10].

*Question 2: what effect do NLP have on listeners?* It is one thing to establish what information is potentially available in a signal, and another to show that receivers actually attend to it. Listeners may also possess sensory biases, in which case, the effect of a perceptual feature may be partly decoupled from the biological information it encodes. For example, humans and many nonhuman animals strongly associate low frequencies [11], high intensity [12] and acoustic roughness [13] with size and formidability, which callers can exploit to achieve acoustic size exaggeration. Ideally, this type of research requires models linking objective acoustic properties with percepts, but human psychoacoustics is a long way from achieving comprehensive perceptual models, and even less is known about the perception of NLP in non-human animals. Fortunately, this is not an insuperable barrier to progress because a link between the presence of NLP in a perceived signal and the listeners' responses can be demonstrated empirically without fully understanding the underlying psychoacoustics, in two ways: with correlational designs or direct manipulation.

The traditional approach has been to compare the listeners’ reaction to otherwise similar calls with versus without NLP in playback experiments (e.g. [14–16]; the dark yellow arrow in figure 1). Apart from methodological simplicity, this approach has the further advantage that natural calls are used directly for playback, ensuring maximum ecological validity of the stimuli. The main drawback is that it is difficult to infer causality because the presence of NLP in natural vocalizations is strongly associated with other voice characteristics, especially with high intensity and *f*_o_ [13,17]. Even if the stimuli with and without NLP are carefully matched on other relevant acoustic characteristics (e.g. as in [16]), it is difficult to ascertain that listeners attend specifically to NLP. Thus, a more powerful solution for inferring causality is to manipulate NLP experimentally (i.e. to add NLP to a recording, remove NLP episodes or change their type) while preserving all other acoustic characteristics of a vocalization (the green arrow in figure 1). It is desirable to repeat the manipulation in a wide range of stimuli that vary in their duration, *f*_o_ range, caller characteristics such as sex and age, etc., to ensure that the results generalize to a broad range of vocalizations and to increase the statistical power, which depends both on the number of stimuli and the number of times each stimulus is evaluated [18]. Accordingly, perceptual studies of NLP require tools that can manipulate them in recordings with high precision and flexibility, and preferably in a user-friendly framework that will streamline the creation of many stimuli.

In this paper, we aim to provide an up-to-date review of the analytical techniques and practical tools for working with NLP in the context of these two research questions. We begin with a discussion of the simplest and most common approach to NLP detection—manual NLP annotation—highlighting its pitfalls and offering possible solutions and complementary measures such as general acoustic descriptives. We then consider each NLP type in turn, discussing suitable methods for their analysis and experimental manipulation. Given the intended research application, we do not cover all possible methods of creating NLP (e.g.,with biomechanical/computational models of phonation), but focus specifically on their *manipulation* in recordings. Within the scope of this text, the term ‘manipulation’ is intended to be an unbiased and non-judgemental way to refer to specific alteration of acoustic signal, namely adding or removing NLP episodes or changing their type in recorded or synthesized vocalizations that are naturalistic enough for playback experiments. The key theoretical considerations and algorithms are covered in the main text at a conceptual level, but we also provide the datasets and complete code for all presented examples and simulations in the electronic supplementary material [19].

## 2. NLP annotation and quantification

**Figure 2.**
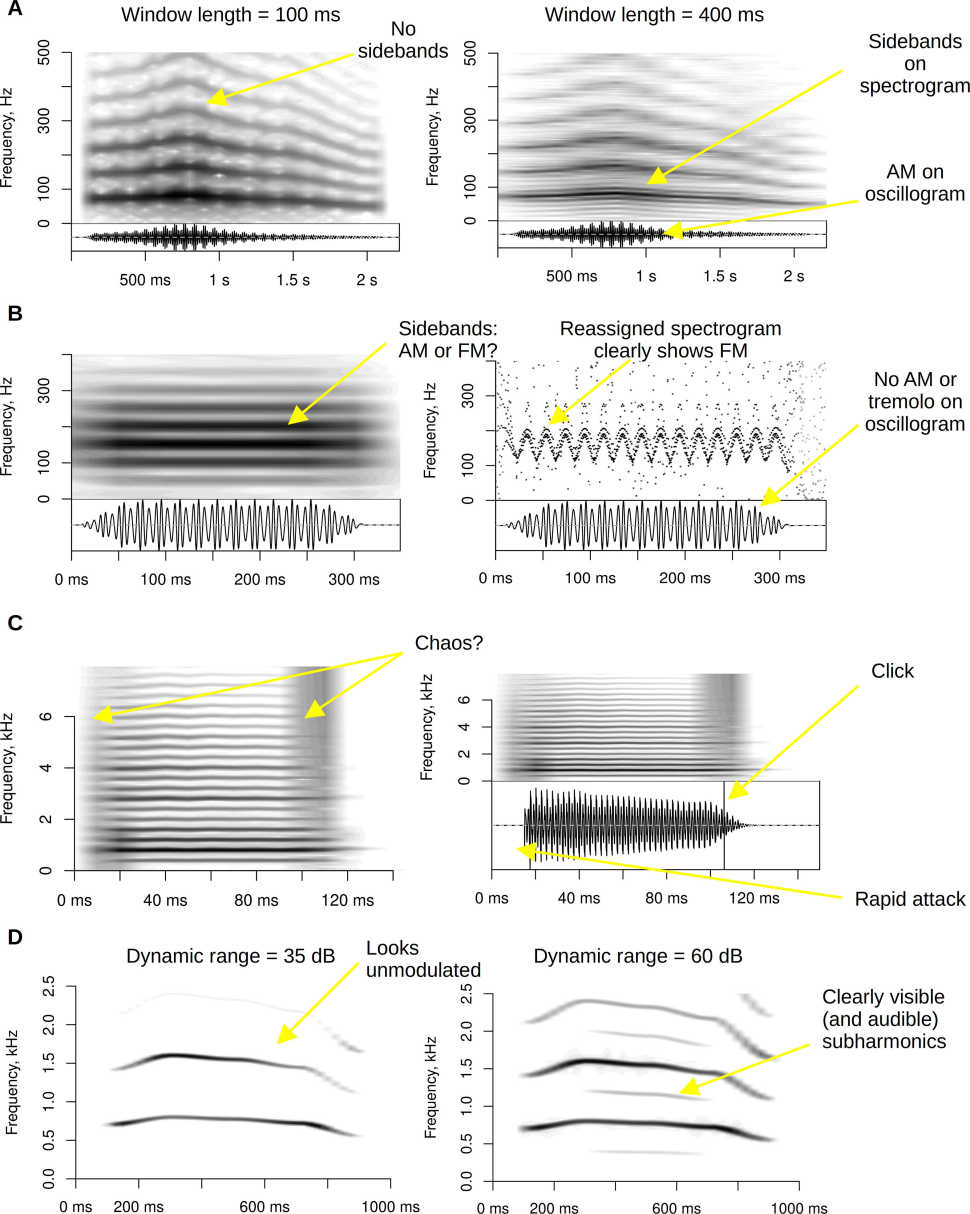
Visualizing NLP: some common problems and solutions.

NLP analysis in bioacoustics typically begins with researchers manually annotating NLP episodes by means of listening to each recording and either inspecting the raw (acoustic) signal in the time domain or scrutinizing some sort of signal feature visualization. The most common of these is the spectrogram, but a number of other visualization tools exist (the key methods and software options are listed in electronic supplementary material, table S1). When it comes to classifying the different NLP, this approach is time-consuming and often highly subjective, potentially suffering from several issues:

1. Implicit or explicit variation of parameters for display generation has a major impact on NLP detection. For instance, variation of the spectrogram’s dynamic range (typically not reported in scientific publications) can be critical for whether subharmonics are discernible in the generated spectrogram or not (figure 2D). Misclassification of NLP can also be due to imperfections and artefacts in the analysed recordings such as aliasing, clipping or reverberation [20].
2. As soon as an experimenter listens to a sound to decide whether NLP are present, two sorts of biases are potentially introduced. The first is an individual bias, which depends on the experimenter’s previous experiences and training. The second is an overall anthropocentric bias. In most cases, very little is known about NLP perception in the investigated species, and there is no guarantee that the features identified by human listeners are relevant to the studied animals. For example, humans are unusually good at pitch discrimination compared to many other mammals [21], potentially making minor frequency jumps in mammalian calls more salient to us than they would be to conspecifics. In contrast, high-frequency biphonation in dog whines may not even be audible to humans [22], not to mention NLP in ultrasonic calls like rodent vocalizations, which can only be detected visually or by transposing the recordings several octaves down in frequency.
3. Finally, both manual and automatic detection of NLP would require a consensus among the bioacoustic research community concerning NLP classification, as well as a standardized approach to their assessment and interpretation. Despite notable efforts, such as signal typing conventions proposed for the human voice [23,24] and the NLP workshop in Saint-Étienne (2023) that led to this special issue, so far there is no consensus regarding even the nature of NLP and their basic types, much less the best approach to their detection and analysis.

For all these reasons, manual NLP annotations do not necessarily correspond to the ground truth of vocal production, or even of NLP perception in nonhuman species. Nevertheless, given its feasibility, manual annotation remains the go-to approach in NLP research [25–31], so it would be important to ascertain how well it captures the reality of vocal production. In the absence of suitable datasets for doing so, we tested at least the inter-rater reliability with which several trained raters performed manual annotation of NLP episodes. Specifically, we asked the attendants of the NLP workshop in Saint-Étienne in June 2023 to note all NLP episodes in a randomly selected subset of 23 vocalizations from a published corpus, all of which were reported as containing some NLP in the original publication [32]. The recordings included 10 human nonverbal vocalizations (5F + 5M), 10 speech samples (5F + 5M) and three samples of a cappella singing (2F + 1M); the duration varied from 2 to 10 s. Ten raters independently annotated four NLP types: frequency jumps (sudden changes in the fundamental frequency), sidebands (additional spectral components around each harmonic), subharmonics (additional spectral components at a rational fraction of the fundamental frequency) and chaos (irregular, noisy phonation). We then calculated the agreement between all possible pairs of raters about the status of each 100 ms frame. The average agreement was 80% for the presence or absence of NLP per frame, 60% for NLP type (excluding frequency jumps, which have no duration) and 60% for the presence of a frequency jump within ± 50 ms of one annotated by another rater (see vignette *manual_annotation* in the electronic supplementary material [19] for the complete analysis of inter-rater agreement). Thus, highly trained and motivated raters are reasonably consistent at detecting NLP episodes in audio recordings of human voice (sidebands, subharmonics or chaos), but the classification of NLP types appears to be less reliable. Crucially, manual annotations have better internal validity as measures of *perceived* nonlinearities or general harshness in the voice (especially when working with human voices), making them more suitable for research on NLP perception rather than NLP production.

To ensure that the results of manual annotation are as accurate as possible, it is important to avoid a few common pitfalls. Given the importance of the chosen visual representation (figure 2), it is helpful to compare several approaches and settings. In particular, the spectrogram can be juxtaposed with the raw waveform (oscillogram), which is an under-utilized, but very informative medium for detecting NLP such as slow amplitude modulation (figure 2A). The spectrogram itself may need to be adjusted depending on the acoustic characteristics of each analysed fragment. Short windows are good for resolving rapid transitions that could otherwise be blurred (e.g. frequency jumps) or misclassified as NLP (e.g. rapid frequency sweeps in bird songs, which may appear as sidebands if the window is too long). On the other hand, extremely long windows upwards of 400−500 ms may be necessary for visualizing stable, but very low-frequency amplitude modulation in calls such as alligator bellows [33]. It may also be helpful to try less familiar visual representations such as time-frequency reassigned spectrograms or wavelet-based transforms [34,35], phasegrams [36], and modulation power spectra [37,38]. These approaches are demonstrated in vignette *visualization* in the electronic supplementary material [19], and we provide ready-to-use R code for their implementation.

Quantitative analysis of acoustic signals is often used as a complement to manual annotation, or even as the only feasible approach when the analysed corpus of recordings is very large. This saves time, dispenses with the need for trained raters, and the results are both objective and reproducible. The main drawback is that most measures are only indirectly affected by NLP (‘General acoustic measures’ in electronic supplementary material, table S1), and individual metrics are typically affected by a number of NLP in a complex fashion. For example, a drop in signal periodicity (e.g. as measured by the harmonics-to-noise ratio [HNR]) might be due to NLP or some other causes (background noise, breathy phonation, etc.), and this lack of specificity can have major implications for the substantive interpretation of obtained results. In addition, most software for voice analysis, such as the popular open-source toolbox Praat [39] and its algorithms, is designed for analysing nearly periodic signals, typically speech. Other measures, discussed in the sections on each NLP type below, are more theoretically grounded, being derived from nonlinear dynamics (‘Nonlinear time series analysis’ in electronic supplementary material, table S1), or designed to capture particular NLP types (‘NLP-specific acoustic measures’ in electronic supplementary material, table S1).

As an exemplary check of NLP specificity, we calculated a variety of acoustic features (generic, NLP-specific and derived from nonlinear time series analysis), frame by frame, in 5000 fully synthetic vocalizations (with ground truth of NLP presence and type known *a priori*), as well as in 1518 audio recordings of human nonverbal vocalizations, singing and speech from [32] with a total duration of two hours and nearly 300 000 overlapping STFT frames 50 ms each (with NLP annotated manually). We then compared the values of each acoustic feature in STFT frames depending on the presence and type of NLP (see vignette *analysis_any-NLP* in the electronic supplementary material [19]). The main conclusion was that the presence of NLP explained vastly more variance of the analysed acoustic measures in synthetic sounds compared to annotated recordings, which could indicate that manual NLP annotations are not entirely accurate (as suggested by the analysis of inter-rater reliability above), and/or that real-life recordings are too ‘messy’ for this kind of acoustic analysis to pick up NLP-specific features. Two measures, the amount of amplitude modulation in the ‘roughness’ frequency range [40] and cepstral peak prominence (CPP), appeared to be most robust to noise in real recordings. Notably, however, none of the tested features were really suitable for discriminating between vocalizations with and without NLP, and especially not for distinguishing deterministic chaos in voiced fragments from turbulent noise in unvoiced fragments.

Having briefly considered the difficult challenge of annotating NLP manually or using proxy acoustic measures, we now turn to the algorithms that have been designed for analysing and manipulating specific NLP types.

## 3. Frequency jumps

Sudden changes of *f*_o_, known as frequency jumps or pitch jumps, have primarily been researched in the context of human singing [41–43], but they are also found in a variety of animal calls [16,44,45] and in human nonverbal vocalizations such as screams [32,46] and baby cries [47]. Their possible causes include both conditions intrinsic to the vocal folds [48,49] and source–filter interaction with the resonances of either the supralaryngeal vocal tract or the tracheal vocal tract [50–54] (see [55] for more details). The best understood example is the transition between vocal registers in human singing, often between the modal or chest voice and the falsetto [43,56,57]. Such voice breaks can occur in both upward and downward directions [41,43,44,48,53,58,59]. Crucially, frequency jumps during register transitions are not merely rapid *f*_o_ glides: the larynx suddenly transitions into a different vibratory mode, often with brief episodes of subharmonics or other nonlinearities during the transition [43,49]. Thus, true frequency jumps constitute bifurcations and should be considered a type of NLP.

### (a) Analysis

Sophisticated algorithms have been tested in human voice science to detect frequency jumps (often in the context of register transitions) from the electroglottographic signal (EGG) based on statistics such as sample entropy [42,56,60]. In practice, frequency jumps are often annotated manually based on inspecting narrowband spectrograms, and perhaps also listening to the recordings, although the exact method is seldom specified [16,32,45]. As with all NLP, this introduces subjectivity in the analysis, particularly when human listeners annotate the vocalizations of other species because of potential differences both in voice production mechanisms and in the perception of pitch discontinuities. A further challenge is posed by rapid *f*_o_ variation caused by super-fast muscles in some animal species that might—without proper analysis tools—be mistaken for a frequency bifurcation [61].

To ensure reproducibility and make the analysis more objective, could frequency jumps be detected automatically? Despite the apparent simplicity, even basic *f*_o_ detection is not a trivial task in itself [62]. Furthermore, once *f*_o_ contours are extracted, and assuming that these are correct, it is not always obvious what constitutes a discontinuity. An algorithmic jump detector might look for *f*_o_ changes in continuously voiced fragments that exceed the average rate of *f*_o_ change before and after the focal frame by a certain threshold. We compared the results of such automatized analysis with manual annotation of frequency jumps and obtained a rather poor match (see vignette *analysis_freqJump* in the electronic supplementary material [19]). As noted above, inter-rater agreement was also far from perfect for frequency jumps. An additional difficulty is that frequency jump detection requires good time resolution of *f*_o_ tracks, making tracking errors more likely and time-consuming to correct manually.

### (b) Manipulation

**Figure 3.**
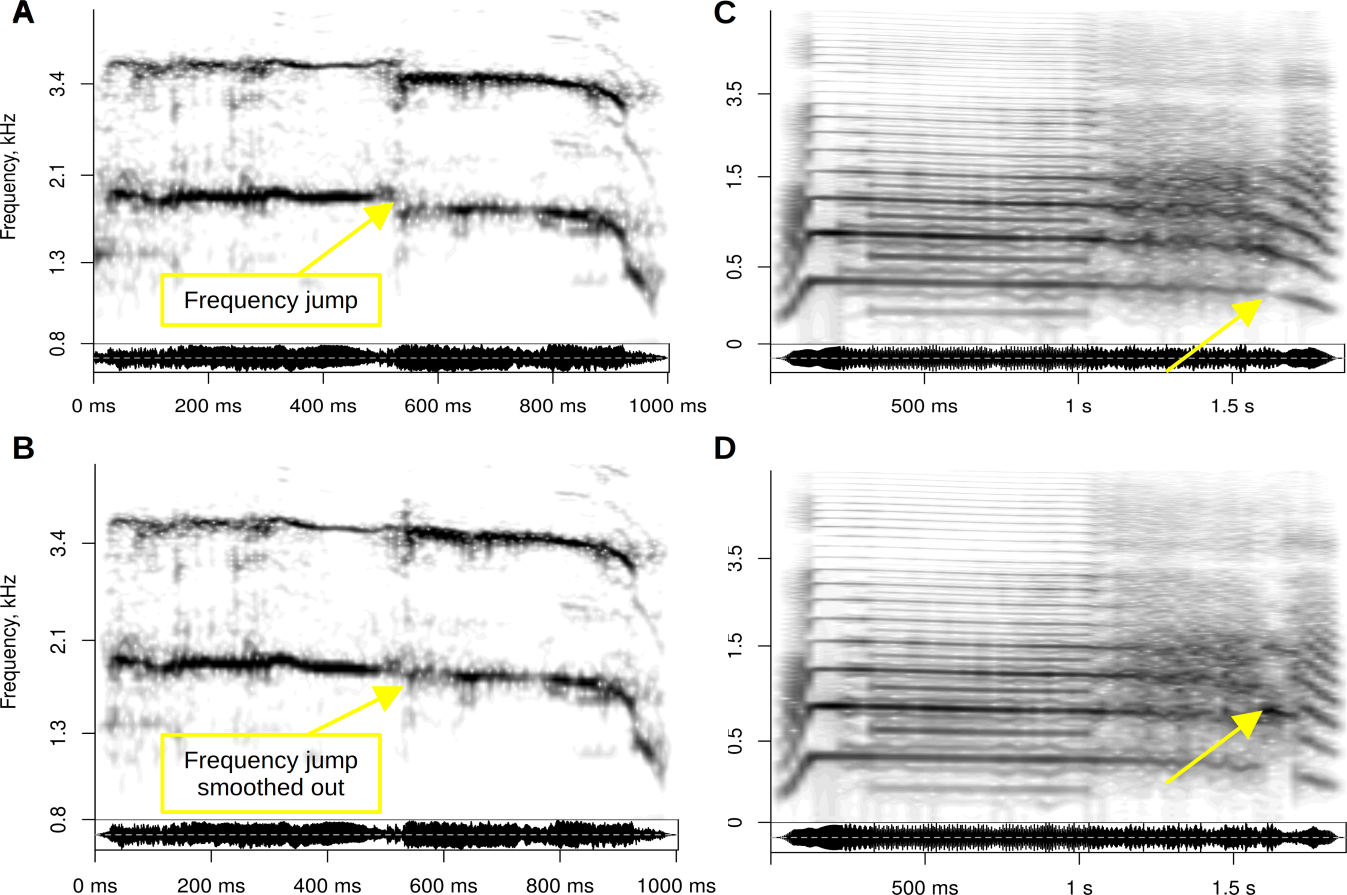
Manipulating frequency jumps in (A,B) recorded (C,D) and synthetic vocalizations. The original recording of a woman’s scream shown in panel A was pitch-shifted with a phase vocoder to smooth out the frequency jump at ~520 ms from 1880 to 1750 Hz in panel B. The human roar shown in panels C and D is fully synthetic, making it straightforward to add or remove two rapid frequency jumps at ~1.5 s without affecting other acoustic characteristics.

Frequency jumps are relatively straightforward to manipulate (add, remove or modify) in recordings of human voice or animal vocalizations using high-fidelity pitch-shifting algorithms that can modify *f*_o_ in existing recordings, while preserving other spectral properties such as vocal tract resonances [63]. The two most common approaches are pitch-synchronous overlap-and-add (e.g. in *Praat* [39]) and phase vocoding (e.g. in the CLEESE toolbox [64]), which operate in the time domain and frequency domain, respectively. Manipulation of existing recordings is preferable when the required fundamental frequency shift is relatively small because this method preserves all other characteristics of the original recording (figure 3A,B), but it may not work very well when the jumps are numerous and/or large. Voice synthesis offers more control (figure 3C,D) and is less likely to introduce artefacts, but it may require more work unless the calls are acoustically very simple (e.g. short pure tones).

**Figure 4.**
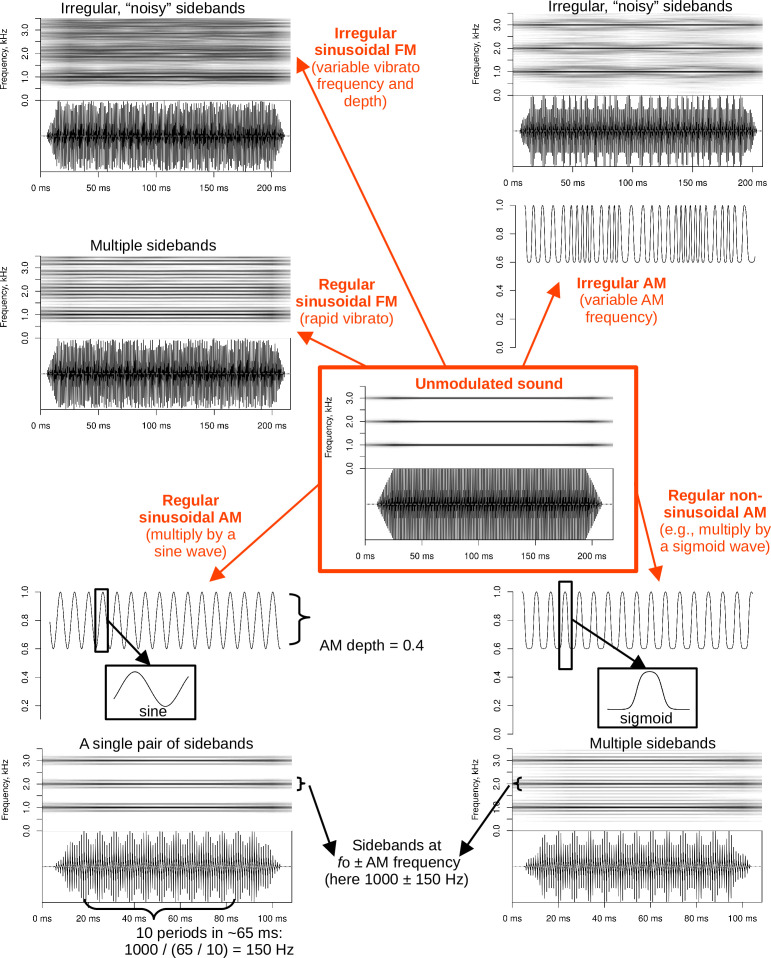
Signal modulation produces a wide variety of sidebands about the harmonics of *f*_o_. A steady harmonic sound with an *f*_o_ of 1 kHz is amplitude- or frequency-modulated; all spectrograms use a window length of 50 ms. See also vignette *synthesis_AM-FM* in the electronic supplementary material [19].

Whichever method is used, the manipulated vocalizations should sound natural, which is not merely a matter of avoiding artefacts of (re)synthesis [63], but also of ensuring a plausible spectro-temporal context for each jump guided by natural recordings and the insights gained from biomechanical modelling. For instance, *f*_o_ would normally jump upward during an ascending frequency sweep, and vice versa (e.g. see [52], fig. 4), ideally with a naturally sounding concomitant change in amplitude and voice quality. It may thus be safer to find a recording with a frequency jump and to remove it by smoothing out the *f*_o_ contour, rather than to introduce a new frequency jump where there was none originally (the same reasoning applies to other NLP as well). Finally, the average *f*_o_ of a recording should not be greatly affected by manipulating frequency jumps, which would otherwise constitute a confound. For instance, the elimination of a frequency jump in figure 3A,B has no effect on the mean *f*_o_, whereas the manipulation of frequency jumps in figure 3C,D changes the mean *f*_o_ from 420 to 440 Hz, which should perhaps be compensated for by means of slightly adjusting *f*_o_ in the preceding fragment.

Despite being relatively simple to manipulate, frequency jumps are arguably the least understood NLP outside human singing, having been experimentally investigated in only a few bioacoustic studies [46,65–67]. More studies with direct manipulation of frequency jumps are needed to shed light on their communicative significance.

## 4. Low-frequency amplitude and frequency modulation

Modulation can be of two basic types. Frequency modulation (FM) corresponds to cyclic changes of *f*_o_ itself; a familiar example is FM in the range of 4−8 Hz in classical Western singing, known as *vibrato* [68]. Amplitude modulation (AM, related to musical *tremolo*) corresponds to cyclic changes of the waveform amplitude envelope, as when a trilled /r/ modulates the airflow. At least in human singing, vibrato (FM) is typically accompanied by some amplitude modulation (AM), so in practice these two phenomena are often present simultaneously [69,70]. In terms of system dynamics, modulation turns a limit cycle in the phase space into a torus [20,71,72]. Looking at a spectrogram, both AM and FM can produce sidebands around each *f*_o_ harmonic if the modulation frequency is much lower than *f*_o_ (figure 4).

The modulation frequency can be independent of *f*_o_ or coupled with it in some rational fraction such as 3 : 2 or 2 : 1, in which case we speak of subharmonics instead of modulation [73]. To complicate matters further, irregular AM with variable frequency and amplitude does not create visible sidebands, but simply produces a noisy-looking spectrogram and an irregular, harsh-sounding voice quality that may look and sound similar to chaos (figure 4). For example, using high-speed imaging, Borch *et al*. [74] showed that rock singers can voluntarily make supralaryngeal mucosa vibrate, creating either subharmonics or irregular AM even when the vocal folds are vibrating in a nearly periodic fashion. Furthermore, the same production mechanism—simultaneous vibration of aerodynamically coupled vocal folds and supralaryngeal structures such as the ventricular folds—can also create true deterministic chaos [75]. In sum, low-frequency modulation is a rather complex and vaguely defined, albeit very common, NLP category. Bioacousticians often label anything that produces visible sidebands simply as *sidebands*, remaining agnostic as to the underlying mechanism.

### (a) Analysis

FM is relatively straightforward to visualize on a spectrogram and to measure if it is slow relative to *f*_o_. For a slow sinusoidal FM, the frequency can be estimated directly from a spectrogram as the reverse of a single period of *f*_o_ oscillation (5 or 10 consecutive periods can be averaged to improve the precision). When conceptually considering a nonsinusoidal FM, the spectrum of the *f*_o_ contour can be searched for spectral peaks that correspond to FM frequencies. For instance, Herbst *et al*. [73] found two such peaks—two dominant frequencies—in the vibrato produced by Freddie Mercury. Two main metrics are typically computed: modulation rate (the vibrato frequency) and modulation extent (the vibrato amplitude). Depending on the method of visualization and FM frequency, FM can visually appear either as vibrato (slow FM, short analysis window) or as sidebands (fast FM, long analysis window). If FM becomes very rapid relative to *f*_o_, the individual cycles of modulation can no longer be resolved with a standard spectrogram because the instantaneous frequency changes too much within an analysis window (as this window must be kept long enough to resolve the *f*_o_ itself). As a result, the spectrogram of a signal with rapid FM will show sidebands around *f*_o_ harmonics instead of vibrato, making the situation visually indistinguishable from AM (figure 4). For instance, a conventional spectrogram with a window length of 100 ms in figure 2B shows strong and stable sidebands, whereas a time-frequency reassigned spectrogram with a window length of 10 ms still captures the 50 Hz FM while preserving reasonable frequency resolution (a conventional spectrogram with a 10 ms window cannot even resolve the *f*_o_ itself at 150 Hz). Perceptually, rapid FM also no longer resembles vibrato; for instance, the tone in figure 2B sounds like a steady, unmodulated note with the same pitch as FM frequency (50 Hz).

As for amplitude modulation, the easiest method of estimating AM frequency is to measure the period of one or several adjacent modulation cycles on an oscillogram (figure 4). More formally, AM can be measured directly from the amplitude envelope (e.g. from the magnitude of the analytical signal obtained with the Hilbert transform) or from the modulation spectrum, which is a two-dimensional Fourier transform of the spectrogram [37,38]. Both methods are demonstrated in vignette *analysis_amDep* in the electronic supplementary material [19]. In our simulations based on synthesizing and analysing 10 000 vocalizations with known AM frequency and depth, AM frequency estimates derived from the envelope were found to be less reliable when the true modulation frequency dropped close to the lower end of the analysed frequency range, whereas the modulation spectrum estimates erred when AM was too rapid relative to the window length used to create the modulation spectrum. The two methods can thus be combined to capture a wider range of AM frequencies. An important practical tip is to narrow down the range of considered AM frequencies as much as possible, based on what is known about the species’s biology and vocal behaviours.

### (b) Manipulation

Slow, vibrato-like FM can be introduced in a recording using the same pitch-shifting techniques that were described above for frequency jumps. Singing voice synthesis is also routinely performed with a controlled amount of vibrato (e.g. [76–78]). Rapid FM is an uncommon manipulation to perform on an existing recording because pitch-shifting algorithms can introduce artefacts at high modulation rates, but it is achievable with parametric voice synthesis. AM is probably the easiest type of NLP to add to a recording: all that is needed is to multiply the sound by a modulating waveform ranging from (*1—AM depth*) to 1. If the modulating waveform is a pure sine, which may be uncommon in animal vocalizations, it creates a single pair of new harmonics at *±AM frequency* around each partial of *f*_o_. More complex, nonsinusoidal AM creates multiple harmonics at *±AM frequency × integer*, which form characteristic sidebands around each *f*_o_ partial (figure 4). This is a popular method for creating rough-sounding voices, which can even be applied in real time [64,79]. The manipulation of AM and FM is demonstrated together with subharmonics in vignette *synthesis_AM-FM* in the electronic supplementary material [19].

## 5. Subharmonics

Subharmonics are additional frequency components (*f*_sub_) at a rational fraction of *f*_o_, typically at *f*_sub_ = *f*_o_/2 or *f*_o_/3, but potentially more complex situations are possible such as *f*_o_ : *f*_sub_ = 3 : 2 [80,81]. They can be produced by partly desynchronized, but still strongly coupled vocal folds or parts thereof that vibrate at harmonically related frequencies, which can be caused by the entrainment of two vibratory modes of the vocal folds [81–83], asymmetric tension on the two vocal folds [84–87], or source–filter interactions with supraglottal [51] or subglottal [53] resonances. Another possible origin of subharmonics is simultaneous frequency-locked vibration of two oscillators such as the vocal folds and the ventricular folds [73,88–90] or aryepiglottic folds [91]. When subharmonics are caused by AM, the modulation depth can be defined as the difference in the amplitude of adjacent glottal cycles expressed as a proportion of the sum of the two amplitudes; in the case of FM, it is defined as the difference in the periods instead of amplitudes of adjacent cycles [92,93]. Amplitude and frequency modulation is thus a more general phenomenon, of which subharmonics are an important and common special case [92].

### (a) Analysis

Subharmonics can be detected and quantified in several ways. One approach is to literally compare, in the frequency domain, the amplitude of spectral peaks corresponding to *f*_o_ partials and subharmonics. The only available open-source algorithm of this kind [94] appears to produce valid estimates of the strength of subharmonics provided that it is properly tuned [92]. Our own simulations suggest that, with realistic noisy recordings, a more robust method is to work with the cepstrum, looking for peaks at a fraction of *f*_o_ (vignette *analysis_subh* in the electronic supplementary material [19]). Obviously, there exists a ‘chicken-and-egg’ problem: *f*_o_ tracking must be accurate, otherwise estimates of subharmonic frequency and depth are meaningless, yet the very presence of subharmonics complicates *f*_o_ detection. One approach is to correct *f*_o_ contours manually prior to analysing subharmonics; another is to label episodes of subharmonics first and to pass on this information to the *f*_o_ tracker. If neither of these options is feasible, it may be safer to exclude this particular signal from the analysis.

### (b) Manipulation

At least three distinct ways of manipulating subharmonics have been described in the literature. The first is to multiply the signal by a low-frequency modulating waveform, which can produce subharmonics if the modulation frequency is kept at *f*_o_/2 (or any other integer rate) at each time point [79]. This method is straightforward to apply to any sound, including instrumental music [95]. The main limitation is that it is critically dependent on accurate *f*_o_ tracking, which can be problematic when working with aperiodic or relatively noisy signals. The second approach is to vary the amplitude and timing of adjacent glottal pulses during voice synthesis [96]. For instance, it is achieved by delaying and attenuating in amplitude every second glottal cycle in the well-known Klatt synthesizer [97]. Finally, a brute-force approach is to literally synthesize new partials spaced by a fraction of *f*_o_ [98]. Like Klatt’s method, this requires parametric synthesis, but the connection between control parameters and the output is more transparent, and any *f*_o_ : *f*_sub_ ratio can be achieved (see vignette *synthesis_AM-FM* in the electronic supplementary material [19]). In particular, the depth of subharmonics is then specified in the frequency domain as the amplitude of subharmonic (*f*_sub_) partials relative to *f*_o_ partials, which is more perceptually relevant compared to time-domain definitions [96].

## 6. Biphonation

Signals with two frequency components that are not phase-locked have phase trajectories in the shape of a torus [99]. Biphonation is commonly distinguished from other two-frequency phenomena by stipulating that both frequencies should evoke pitch sensations (unlike low-frequency modulation), change independently, and not form a rational ratio (unlike subharmonics) [20,71,72]. An additional requirement is sometimes proposed that both frequencies should be produced by a single sound source—for example, by the same half of the syrinx [100]. The term *diplophonia*, common in the human voice literature, does not make such distinctions and includes any two-frequency phenomena [80].

Biphonation *senso stricto*, with two or even three independent audible frequencies, has been reported in human pathological voices [71] and singing [101], as well as in vocalizations of other mammals [22,102–105] and birds [100,106,107]. The two sources may be physically coupled: for example, the higher frequency (*g*_o_) can be amplitude-modulated by the lower *f*_o_, generating complex sidebands [103,104]. On the other hand, two fully independent frequencies may be produced without noticeable coupling, as in bird songs consisting of two independently controlled sound sources in the syrinx [106].

### (a) Analysis

The task of tracking two independent frequencies is challenging and rather exotic in bioacoustics. There are numerous proposed solutions for multi-pitch estimation in polyphonic music [108] and conversation analysis [109], but their robustness and applicability to biphonation remain largely unknown. A rare exception is the work by Aichinger, who developed and tested algorithms for simultaneous tracking of two frequencies in pathological human voices, essentially by means of testing several paths through *f*_o_ candidates [110,111]. Tools for AM analysis may also be applicable when one frequency is many times lower and amplitude-modulates the higher one, but otherwise manual annotation and description based on the spectrogram and the phase space are the only viable approaches at present.

### (b) Manipulation

Biphonation can be created by mixing separate recordings or (re)synthesized versions of two or more vocal sources. The key challenge is that these vocal sources may be coupled: for example, one may be modulating the other, or *f*_o_ and *g*_o_ may be momentarily locking to each other and/or to resonance frequencies. There are some workaround solutions such as adding a multiplicative term when mixing the two sounds in order to achieve modulation (see vignette *synthesis_biphonation* in the electronic supplementary material [19]). Potentially tractable cases for synthesizing biphonation involve fully independent sound sources, such as in bird and whale songs, but there is only limited work in this area [112]. Overall, however, two-frequency calls like horse whinnies [102] or wapiti bugles [103] are some of the most challenging vocalizations to work with.

## 7. Chaos

An entire branch of mathematics, known as chaos theory, has been developed to model the behaviour of deterministic systems that nevertheless display seemingly random or chaotic behaviour. More formally, chaotic systems are deterministic, bounded, aperiodic and sensitive to initial conditions [113]. Belying this apparent complexity, the attractors of chaotic systems can be relatively simple when appropriately reconstructed in the phase space. Applied to vocal production, the term *chaos* designates ‘nonrandom noise’ [51], namely highly irregular vibration of the vocal folds and other coupled structures [114] that is deterministic, being the result of a mechanically simple system with relatively few degrees of freedom, yet seemingly random or noisy. Thus, aspiration noise as in /s/ would not be described as chaos [23] because the source of randomness in this case is turbulence in a very high-dimensional system, and its attractor in low-dimensional phase space is structureless.

### (a) Analysis

Like other NLP, chaos is often annotated manually in audio recordings. There is typically a residual trace of the original *f*_o_ and perhaps even a few of the lower harmonics on the spectrogram, but with a varying amount of spectral smearing, making chaotic phonation look superficially similar to turbulent noise (e.g. whispered speech). Furthermore, given the limitations of spectrograms in terms of the tradeoff between time and frequency resolution, they are potentially inappropriate for distinguishing between chaos and irregular AM/FM. Given its similar appearance, chaos is also difficult to distinguish visually in the time domain from noise or signals containing FM. Therefore, inspection of the waveform is not very helpful, except that it sometimes reveals other causes of chaos-like spectral noise such as rapid sound onsets, clicks or recording artefacts (figure 2C).

The most powerful visual tool for distinguishing between (high-dimensional) noise and (low-dimensional) chaos is the phase space (see vignette *phasegrams* in the electronic supplementary material [19]). Formal mathematical methods of nonlinear time series analysis have also been applied to voice analysis, mostly in the context of quantifying voice pathology (dysphonia). A great variety of visualization aids and measures have been explored, the most popular of which are the correlation dimension D2, Lyapunov exponents and Poincare sections [36,113]. Some measures may be more noise-robust and tolerant of nonstationary signals: for instance, the correlation dimension D2 is claimed to tolerate noise levels of 8% or even up to 20%, and to perform robustly with relatively short voiced frames of only 20 ms in duration [115]. D2 was found useful for detecting voice pathology in several studies [115,116]. For instance, it was elevated in patients with vocal tremor caused by Parkinson’s disease or vocal polyps [117] and moderately correlated with subjectively rated dysphonia [118]. D2 and the largest Lyapunov’s exponent also discriminated between normal and irregular phonation in excised larynges [119–121]. However, other work suggests that D2 is only meaningful in noise-free and relatively periodic signals [122], making it less suitable for detecting episodes of chaos in field recordings. In fact, D2 and shimmer were reported to be elevated in a cappella opera singing compared to other musical genres, possibly because of the vibrato [123]. Accordingly, even when dealing with high-quality recordings of steady vowels, some authors recommend classifying the voices manually into three or four types, from mostly periodic to fully aperiodic, and excluding the unsuitable voice types from the analyses of *f*_o_ perturbation and nonlinear measures [23,24], which brings us full circle back to manual classification and annotation.

Our own simulations suggest that D2 is indeed among the most robust measures derived from nonlinear dynamics, but still far from a reliable ‘litmus test’ for chaos in relatively noisy real-life recordings (see vignette *analysis_any-NLP* in the electronic supplementary material [19]). Computationally cheaper measures include summaries of Poincare sections such as their Shannon entropy or the phasegram complexity estimate, which is calculated as the one-dimensional correlation dimension along each Poincare section [114]. An important direction for future work would be to better validate D2 and related nonlinear measures for detecting NLP in various species because most research to date has focused rather narrowly on diagnosing human voice pathology.

Tools for nonlinear time series analysis can be found in specialized software like TISEAN [124] and in various R libraries (see electronic supplementary material, table S1 for details). An important proviso is that the effective use of these tools requires advanced mathematical expertise for diagnostics and customization. An additional—and often neglected—condition is that the input for nonlinear analysis is supposed to be a noise-free and stationary signal, where the mean and variance do not change over time. Real-life recordings like continuous speech or nonverbal vocalizations routinely violate the key assumptions of nonlinear analysis, being relatively short, noisy and nonstationary—for example, *f*_o_ can noticeably change within an analysis window [113,118]. In particular, just like perturbation measures (jitter and shimmer) are not meaningful when *f*_o_ cannot be detected, nonlinear dynamic analysis is not applicable to high-dimensional noise of the kind found in breathy or whispery voices [23] and in recordings with a low signal-to-noise ratio.

Since the phase space is often reconstructed by plotting the signal against its time-delayed copy, another challenge is to determine the optimal time lag when the signal is no longer periodic. This lag is normally set to approximately 1/4 of the fundamental period to reduce the correlation of the original and time-delayed versions of the signal, and the optimal value can be estimated from the autocorrelation or mutual information function [113]. Alternatively, we can perform a Hilbert transform and plot its real versus imaginary components [36,60]. Because the phase of each frequency component is shifted by ±*π*/2, this decorrelates the components and produces a suitable phase space without the need to determine *f*_o_, which is nearly impossible during episodes of chaos (see vignette *phasegrams* in the electronic supplementary material [19]).

### (b) Manipulation

Deterministic chaos in animal vocalizations is sometimes imitated simply by inserting a short episode of white noise into a tonal call [125,126]. There is some evidence that birds respond similarly to synthetic chaotic time series and white noise [127]. However, chaos found in natural calls retains residual periodicity and formant structure, making it different from both white noise and mathematically constructed chaos such as the output of a logistic map. We have therefore tested an alternative method of imitating chaos by means of stochastically perturbing the periodicity of a vocal source. Because of the stochastic implementation, this is not true low-dimensional deterministic chaos, but it has been shown to create a perceptually adequate imitation thereof in human speech [128], nonverbal vocalizations [31,46,129,130] and animal calls [66]. As implemented in *soundgen* [98], this is achieved by adding strong and very rapid Gaussian jitter to *f*_o_. This works for any *f*_o_ and jitter depths because, in contrast to most algorithms for voice synthesis, jitter in *soundgen* corresponds to perturbing the instantaneous frequency repeatedly within glottal cycles rather than resetting it at cycle boundaries [131]. Alternatively, random and very rapid jumps of the instantaneous frequency (again, not synchronized with cycle boundaries) can be added between two *f*_o_ values: a latent *f*_o_ contour and another value such as a nearby formant. This is based on the observation that chaotic behavior is often brought about by an interaction between two or more oscillation modes [114], which can be activated simultaneously and superimposed [132]. Examples of both approaches are demonstrated in vignette *synthesis_chaos* in the electronic supplementary material [19].

## 8. Conclusions

There is mounting evidence that NLP encode a wealth of biologically important information about the caller, from individual identity to emotional state, and that listeners carefully attend to these acoustic features. In order to unlock the potential of NLP for a better understanding of vocal communication in the animal world and in human societies, it is crucial to have effective, accurate and user-friendly tools for working with these acoustic phenomena. In this methodological review, we provide a necessarily brief, but relatively comprehensive description of modern techniques for NLP analysis, as well as for their manipulation and perceptual testing in playback experiments.

When it comes to NLP analysis, many challenges and exciting opportunities still lie ahead. To make claims about the presence of NLP, their episodes must be annotated, but both manual and automatic approaches have their particular drawbacks. Even when performed by trained researchers, the annotation task is far from trivial, and we describe several common pitfalls and suggest solutions for improving its accuracy. Several automatically extracted acoustic descriptives do capture some of the NLP-related variation in voice quality, but these are typically not specific enough to infer the presence of NLP in general or of their specific types. The mathematical tools of nonlinear time series analysis are potentially useful for detecting chaos, but the measures proposed so far are not sufficiently robust or user-friendly to be of much practical use, with the possible exception of the correlation dimension D2 and the use of the phase space as a visual aid. This calls for further work on applied NLP analysis and a better consensus on the best practices in the scientific community.

The domain of applied NLP manipulation and synthesis appears to be more mature as several effective experimental techniques have now been developed and validated. One major tool to exploit is pitch-shifting algorithms, which can create frequency jumps and frequency modulation with high precision, sometimes even in real time. Amplitude modulation and subharmonics are also relatively straightforward to add to a recording. Voice synthesis is the ultimate tool for the most demanding NLP manipulations, including chaos. A major advantage of parametric synthesis is the ability not only to add a specific NLP, but also to remove it. A promising approach is to find prototype recordings with various NLP and then resynthesize them for playback experiments, either removing the NLP completely or modifying their type (e.g. turning an episode of chaos into subharmonics). With this method, the manipulated NLP will appear in their natural spectro-temporal context, greatly improving the ecological validity of manipulated stimuli compared to simply inserting NLP in an arbitrarily chosen location in a recording that originally did not contain NLP at all.

In future, the work on NLP detection and manipulation can greatly benefit from better theoretical and methodological integration between clinical voice research and bioacoustic or psychological studies aiming to understand the communicative function of NLP. A particularly welcome development would be the release of freely available, extensive and well-documented audio collections covering various NLP in a range of species. This will facilitate further methodological advances and provide suitable training datasets for machine-learning algorithms. NLP detection and annotation, in particular, is a natural task for neural networks—with the important proviso that the training data must be valid. Because expert annotations of specific NLP types in audio recordings may not be entirely reliable, the training data should ideally be more objective (e.g. EGG or high-speed imaging) if the goal is to understand vocal production. On the other hand, expert annotations can provide highly valid training data for algorithms whose aim is to capture the perceptual salience of NLP rather than the precise mechanism of their production. Psychoacoustic modeling of NLP perception is another major area for future research, particularly in nonhuman animals. We hope that this special issue will catalyse the multifaceted field of NLP research, promoting better integration and data sharing between different disciplines and research groups.

## Data Availability

Supplementary tables, vignettes, datasets, and R scripts for acoustic analysis and synthesis can be downloaded from the Open Science Framework [19] and online at [133].
